# Implementation of the uterine fibroids Option Grid patient decision aids across five organizational settings: a randomized stepped-wedge study protocol

**DOI:** 10.1186/s13012-019-0933-z

**Published:** 2019-09-02

**Authors:** Peter Scalia, Marie-Anne Durand, Rachel C. Forcino, Danielle Schubbe, Paul J. Barr, Nancy O’Brien, A. James O’Malley, Tina Foster, Mary C. Politi, Shannon Laughlin-Tommaso, Erika Banks, Tessa Madden, Raymond M. Anchan, Johanna W. M. Aarts, Priscilla Velentgas, Joyce Balls-Berry, Carla Bacon, Monica Adams-Foster, Carrie Cahill Mulligan, Sateria Venable, Nancy E. Cochran, Glyn Elwyn

**Affiliations:** 1 The Dartmouth Institute for Health Policy and Clinical Practice, Geisel School of Medicine at Dartmouth College, One Medical Center Drive, 5th floor, Lebanon, NH 03756 USA; 2 Department of Surgery, Division of Public Health Sciences, Washington University School of Medicine, St. Louis, MO USA; 3 Department of Obstetrics and Gynecology, Mayo Clinic, Rochester, MN USA; 4 College of Medicine and Science, Mayo Clinic, Rochester, MN USA; 5 Department of Obstetrics and Gynecology and Women’s Health, Albert Einstein College of Medicine, Montefiore Medical Center, Bronx, New York, USA; 6 Division of Reproductive Endocrinology and Infertility, Department of Obstetrics, Gynecology & Reproductive Biology, Brigham and Women’s Hospital, Harvard Medical School, Boston, MA USA; 7 Aetion, Boston, MA USA; 8 Department of Obstetrics and Gynecology, Radboud University Medical Center, Nijmegen, Netherlands; 9 National Uterine Fibroids Foundation, Colorado Springs, CO USA; 10 The Fibroid Foundation, Bethesda, MD USA

**Keywords:** Implementation, Shared decision making, Patient decision aids, Decision support intervention, Uterine fibroids, Normalization Process Theory, Consolidated Framework for Implementation Research, Picture superiority, Electronic health record, Co-production

## Abstract

**Background:**

Uterine fibroids are non-cancerous overgrowths of the smooth muscle in the uterus. As they grow, some cause problems such as heavy menstrual bleeding, pelvic pain, discomfort during sexual intercourse, and rarely pregnancy complications or difficulty becoming pregnant. Multiple treatment options are available. The lack of comparative evidence demonstrating superiority of any one treatment means that choosing the best option is sensitive to individual preferences. Women with fibroids wish to consider treatment trade-offs. Tools known as patient decision aids (PDAs) are effective in increasing patient engagement in the decision-making process. However, the implementation of PDAs in routine care remains challenging. Our aim is to use a multi-component implementation strategy to implement the uterine fibroids Option Grid™ PDAs at five organizational settings in the USA.

**Methods:**

We will conduct a randomized stepped-wedge implementation study where five sites will be randomized to implement the uterine fibroid Option Grid PDA in practice at different time points. Implementation will be guided by the Consolidated Framework for Implementation Research (CFIR) and Normalization Process Theory (NPT). There will be a 6-month pre-implementation phase, a 2-month initiation phase where participating clinicians will receive training and be introduced to the Option Grid PDAs (available in text, picture, or online formats), and a 6-month active implementation phase where clinicians will be expected to use the PDAs with patients who are assigned female sex at birth, are at least 18 years of age, speak fluent English or Spanish, and have new or recurrent symptoms of uterine fibroids. We will exclude postmenopausal patients. Our primary outcome measure is the number of eligible patients who receive the Option Grid PDAs. We will use logistic and linear regression analyses to compare binary and continuous quantitative outcome measures (including survey scores and Option Grid use) between the pre- and active implementation phases while adjusting for patient and clinician characteristics.

**Discussion:**

This study may help identify the factors that impact the implementation and sustained use of a PDA in clinic workflow from various stakeholder perspectives while helping patients with uterine fibroids make treatment decisions that align with their preferences.

**Trial registration:**

Clinicaltrials.gov, NCT03985449. Registered 13 July 2019, https://clinicaltrials.gov/ct2/show/NCT03985449

**Electronic supplementary material:**

The online version of this article (10.1186/s13012-019-0933-z) contains supplementary material, which is available to authorized users.

Contributions to the literature

- Our paper will determine the real-world barriers and facilitators to the sustained implementation of patient decision aids.
- We will provide insight on various processes to identify patients eligible to receive a patient decision aid and *how* and *when* to provide these tools to patients to minimize the burden on clinic workflow.
- Ultimately, we will delineate pathways that will enable clinics to determine how their workflow needs to change in order to achieve the sustained implementation of a patient decision aid.

## Background

Uterine fibroids, non-cancerous overgrowths of the smooth muscle in the uterus, develop in nearly half of all women of reproductive age [1]. Some uterine fibroids give rise to heavy menstrual bleeding, pelvic pain, discomfort during sexual intercourse, pregnancy complications, and/or difficulty becoming pregnant [1]. Given racial disparities in the US prevalence of symptomatic uterine fibroids, African-American and Latina women may be more likely to experience these issues and also may have poorer treatment outcomes, greater economic burden, and higher incidence of uterine fibroids in comparison to White women [2–5].

Many options are available to treat uterine fibroids including medications, intrauterine devices (with and without hormones), destruction of the inner layer of the uterine wall (endometrial ablation), removal of the uterus (hysterectomy), removal of fibroids (myomectomy), or reducing blood supply to the uterus (uterine artery embolization). However, insufficient evidence exists about the comparative effectiveness of treatments for uterine fibroids [2, 6, 7]. Choosing the best treatment option for uterine fibroids requires women to consider the unique characteristics of each option and weigh which option might best fit her needs and preferences.

Patient decision aids (PDAs) like Option Grid™ can help educate patients to better navigate preference-sensitive treatment decisions [8, 9]. Option Grids (available in text, picture, or online formats) present evidence-based information in a tabular format, so patients can compare options together with clinicians [10]. Across a variety of clinical contexts, Option Grid PDAs increase patient knowledge and shared decision making without significantly increasing encounter duration [11, 12]. A previous version of the uterine fibroid Option Grid increased patient engagement in the clinical conversation, improved knowledge of treatment options, and increased satisfaction with care [13, 14].

Implementation of PDAs remains difficult across a range of healthcare settings [15]. System and organizational barriers relate to incentives, competing priorities, and established behavior patterns [16, 17]. Despite evidence that it is possible to implement PDAs, particularly if implementation is supported by clinical champions, organizational strategies, and effective electronic health record (EHR) integration efforts, more work is needed to determine how best to routinely adopt these tools in clinical practice [18, 19].

We aim to use a multi-component implementation strategy guided by the Consolidated Framework for Implementation Research (CFIR) and the Normalization Process Theory (NPT) to develop a tailored strategy for each of the five participating gynecology settings to implement the uterine fibroid Option Grid PDA. We will (1) assess each site’s organizational readiness for patient engagement, (2) use a tailored implementation strategy to incorporate the uterine fibroid Option Grid in clinical care at each site, (3) examine the characteristics associated with success and failure to implement and sustain Option Grid use in practice, and (4) integrate new evidence into existing clinical practice guidelines where feasible. We will also evaluate the impact of the implementation strategies on clinical and other relevant outcomes for women across socioeconomic strata who seek treatment for symptomatic uterine fibroids. We hypothesize that providing study settings with various formats of PDAs to help tailor the implementation strategy to their clinic workflow will lead to sustained use of PDAs.

## Methods

### Design

**Fig. 1:**
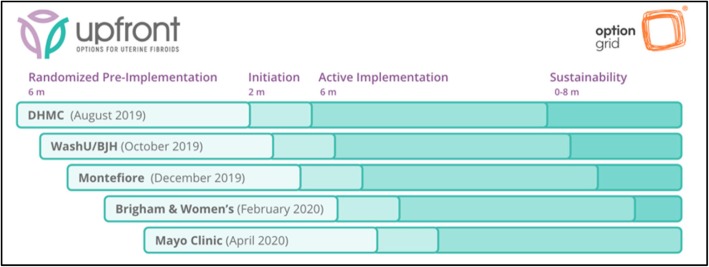
Stepped-wedge study design and timeline

We will conduct a randomized stepped-wedge study where randomization of each site to a pre-implementation start date occurs prior to the start of the study (see Fig. 1 for details). The stepped-wedge design with a baseline pre-implementation phase allows for within-site pre/post comparisons. Following the pre-implementation phase, which occurs at a different time point for each site, we will provide a brief initiation session to introduce Option Grid PDAs and how to use them in practice. After initiation, clinicians will be expected to use the intervention in an “active implementation” phase. The SPIRIT checklist (see Additional file 1) guided our protocol development [20].

#### Theoretical framework

The multi-component implementation strategy is guided by the CFIR and NPT. CFIR is a pragmatic, multilevel framework that guides the evaluation and design of implementation studies [21, 22]. It comprises five domains (intervention characteristics, outer setting, inner setting, characteristics of individuals, and process) and 39 related constructs. These domains interact with one another to influence the effectiveness of the implementation strategy. In the context of our study, CFIR will be used at a macro level to guide implementation by comprehensively addressing four of the five CFIR domains: intervention characteristics, inner setting, characteristics of individuals, and process. Table 1 provides details on the CFIR domains.

**Table 1:** Intervention characteristics that influence implementation success (according to CFIR)

Construct	Short description
I. Intervention characteristics	
A. Intervention source: quality assurance.	EBSCO Systematic Literature Surveillance System (source of evidence for DynaMed Plus product) is best in class.
B. Relative advantage: comparison opportunity	Very few clinicians have used patient decision aids designed to facilitate shared decision making. Our evidence so far indicates that clinical teams are positive about their utility.
C. Adaptability: the degree to which an intervention can be adapted to local needs.	The availability of multiple formats: online and two printed versions (text and Picture Option Grid) allows maximum adaptability to local workflow variations. The online tool can be sent to patients ahead of visits, as well as after visits. The printed versions allow clinicians to tailor the content to local practice variation and to fit the tool into their style of communication with patients of varying literacy and computer literacy levels. We know that clinicians value the ability to add, edit, and make notes on these tools before they give them to patients to take home. The use of pictures maximizes the usability and accessibility of this approach across socioeconomic strata.
D. Trialability: local test	The cost or complexity of using Option Grid is low, and so, we anticipate low resistance to trialability by the participating clinical teams. In other clinical contexts, we have experienced zero resistance to trialability.
E. Complexity: as the number of steps, or the number of people or processes increases, so does the difficulty of implementation.	The Option Grid has been designed to be fast, frugal, and outwardly simple, so that it can fit into decision discussions that will benefit from accurate, accessible information.
F. Design quality and packaging: instills confidence in the intervention.	The EBSCO Option Grid has achieved high quality design with professional user centered graphic design.
G. Cost: investment, supply, and opportunity costs.	Investment will be related to the time taken to learn how to integrate the tool into the clinical workflow, a learning curve that has been observed to take a few interactions. The project will ensure adequate supply; future use will need to ensure online access for sustainability. We anticipate minimal disruption on opportunity costs—clinicians typically cover the type of information in Option Grids. The tool makes the exchange more efficient according to our evaluations. Evidence suggests that Option Grid decision aids do not typically increase consultation time as the content of the tool is information clinicians already provide to patients routinely.
II. Inner setting	
B. Networks and communication: The nature and quality of webs of social networks and the nature and quality of formal and informal communications within an organization.	Eligible patients will be identified using the site’s outpatient scheduling system in advance of their visit. Where possible, the eligible patients will be sent an Option Grid in advance of their appointment and will be instructed to bring the Option Grid to their appointment.
C. Culture: Norms, values, and basic assumptions of a given organization.	We want to help implement a process where patients are engaged in their treatment decisions “upfront” by receiving an intervention that can facilitate a discussion with their clinician regarding their treatment options.
D. Implementation climate: The absorptive capacity for change, shared receptivity of involved individuals to an intervention, and the extent to which use of that intervention will be rewarded, supported, and expected within their organization.	We will assess implementation climate by calculating the expected use of the intervention which is based on the volume of patients visiting each site who have been diagnosed with symptomatic uterine fibroids.
E. Readiness for implementation: Tangible and immediate indicators of organizational commitment to its decision to implement an intervention.	We will determine readiness for implementation from the Measuring Organizational Readiness for patient Engagement (MORE) survey which will be administered prior to the commencement of the pre-implementation phase to 10 stakeholders at various levels of the service delivery team (i.e., clinicians, administrators, managers).
III. Characteristics of individuals	
A. Knowledge and beliefs about the intervention: Individuals’ attitudes toward and value placed on the intervention as well as familiarity with facts, truths, and principles related to the intervention.	ADOPT is a measure of patient attitudes to patient decision aids. Clinicians will be asked to select one or more words that best describes their attitudes to the potential use of patient decision aids from a pool of ten words.
B. Self-efficacy: Individual belief in their own capabilities to execute courses of action to achieve implementation goals.	The words selected by the participating clinicians who complete the ADOPT measure will be indicative of their self-efficacy or the belief in their ability to execute the course of action and achieve implementation goals.
C. Individual stage of change: Characterization of the phase an individual is in, as he or she progresses toward skilled, enthusiastic, and sustained use of the intervention.	We will compare collaboRATE scores (three-item patient-reported outcome measure) before and after the initiation phase to determine individual stage of change.
D. Individual identification with organization: A broad construct related to how individuals perceive the organization, and their relationship and degree of commitment with that organization.	Ten stakeholders at various levels of the service delivery team (i.e., clinicians, administrators, managers) at each site will complete the Measuring Organizational Readiness for patient Engagement (MORE) survey.
E. Other personal attributes: A broad construct to include other personal traits such as tolerance of ambiguity, intellectual ability, motivation, values, competence, capacity, and learning style.	Personal attributes will be determined via the ADOPT survey. Clinicians will circle up to 10 words that will be indicative of their personal traits.
IV. Process	
A. Planning: The degree to which a scheme or method of behavior and tasks for implementing an intervention are developed in advance and the quality of those schemes or methods.	We will be visiting each site multiple times throughout the study to provide support and assess the degree to which each site is willing to adopt our processes.
B. Engaging: Attracting and involving appropriate individuals in the implementation and use of the intervention through a combined strategy of social marketing, education, role modeling, training, and other similar activities.	Our second site visit will aim to attract and involve appropriate individuals in the implementation and use of the intervention. In addition, we will be providing initiation to clinicians to teach them how to use the tools in practice. Each site will also have a “clinical champion”/site principal investigator that will support the implementation of the intervention. The strategy will also include feedback on study processes from the members of the Community Advisory Board.
C. Executing: Carrying out or accomplishing the implementation according to plan.	The primary outcome measure is the number of eligible patients who receive the uterine fibroid Option Grid.
D. Reflecting and evaluating: Quantitative and qualitative feedback about the progress and quality of implementation accompanied with regular personal and team debriefing about progress and experience.	A 23-item instrument—NoMAD Normalization Process Theory (NPT) survey—will be used to capture the perspective of professionals directly involved in the work of implementing the intervention. We will also conduct semi-structured interviews with a convenience sample of clinicians and staff at each of the five clinical sites to identify, monitor, and assess the progression and integration of the intervention and to determine the utility of the Option Grid patient decision aid and the barriers and facilitators to their integration in the clinic workflow. We will also be receiving feedback at our annual site visit to determine the process each site is using to facilitate implementation of the intervention. The outcomes we will be measuring include the extent to which tools are delivered, the extent to which patients are reporting use of Option Grid in appointments, and the collection of collaboRATE scores.

In addition to CFIR’s multilevel approach, NPT will be used at a micro level to focus on the dynamic process that leads to the successful implementation of an innovation in routine clinical practice. NPT is an explanatory theory that helps evaluators understand the process by which organizations embed interventions into their normal work [23, 24]. NPT has four theoretical tenets: (i) coherence, supports individual and collective consensus about an intervention and its purpose; (ii) collective action, the tasks allocated to the various members within the organization to build and sustain use; (iii) cognitive participation, the relational work that influences “implementation and legitimation”; and (iv) reflexive monitoring, the communal appraisal work that aids assessment of the intervention [25].

### Setting

The five study sites are gynecology clinics at (i) Dartmouth-Hitchcock Medical Center in Lebanon, New Hampshire; (ii) Barnes-Jewish Hospital in St. Louis, Missouri; (iii) Montefiore Medical Center in Bronx, New York; (iv) Brigham and Women’s Hospital in Boston, Massachusetts; and (v) Mayo Clinic in Rochester, Minnesota.

### Participants

We will include physicians (attending or resident) who consent to participate in our implementation study and provide care to women with symptomatic uterine fibroids during the project duration at participating sites.

We will include patients showing new or recurrent symptoms of uterine fibroids (e.g., heavy menstrual bleeding, pelvic pressure or pain) who are seeking treatment and meet all of the following inclusion criteria: (i) assigned female sex at birth, (ii) at least 18 years of age, (iii) speak English or Spanish, and (iv) have the ability to complete short surveys online independent of assistance. We will not exclude pregnant patients. We will exclude postmenopausal patients because they may have different treatment options than those presented in the Option Grid PDA used in this study.

### Intervention

To facilitate deliberation about treatment options, the uterine fibroid Option Grid PDA is organized in a tabular format and is based on patients’ frequently asked questions, which were sourced by analyzing the research literature and by qualitative inquiry with stakeholders including gynecologists and 10 women experiencing symptoms of uterine fibroids.

**Fig. 2:**
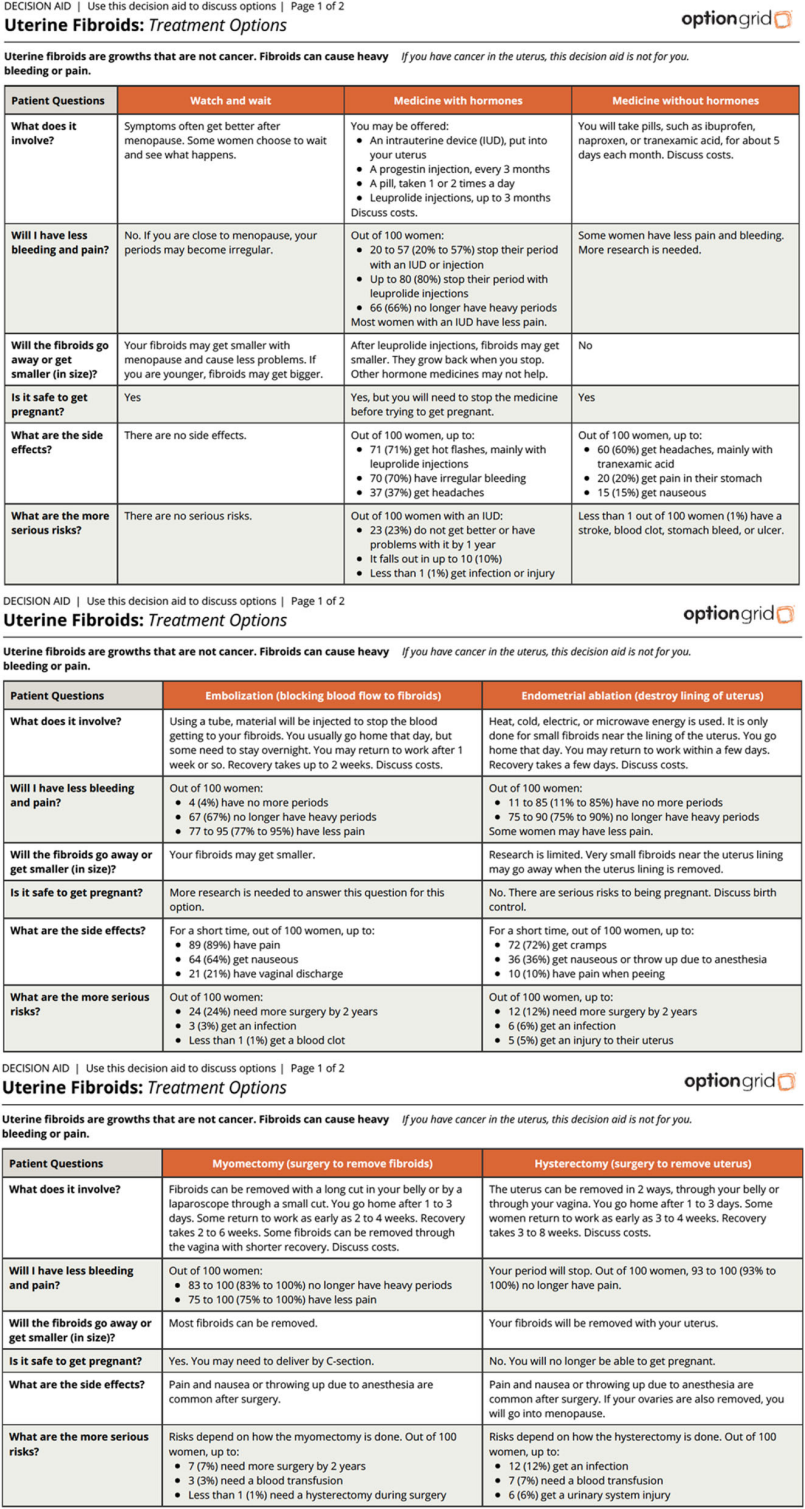
The uterine fibroid Option Grid patient decision aid

The uterine fibroid tool is one of over 22 Option Grid decision aids developed and maintained by EBSCO Health. We helped EBSCO adapt the Option Grid for this study by following a multi-step Community Based Participatory Research (CBPR) approach. The tool (as of May 2019) compares seven treatment options (watch and wait, medicine with hormones, medicine without hormones, uterine artery embolization, endometrial ablation, myomectomy, and hysterectomy) and consists of answers to six frequently asked questions: (1) What does it involve? (2) Will I have less bleeding and pain? (3) Will the fibroids go away or get smaller (in size)? (4) Is it safe to get pregnant? (5) What are the side effects? and (6) What are the more serious risks? The uterine fibroid Option Grid is designed to be accessible to patients across socioeconomic and health literacy strata using plain language (readability of 6th grade or less). See Fig. 2 to view the text version of the Option Grid.

There will be two versions of the uterine fibroid Option Grid available in this trial: (i) text and (ii) text with pictures (Picture Option Grid). The Picture Option Grid uses the same evidence as the text version while integrating images, thus utilizing pictorial superiority to help patients better understand and remember information [26]. Both the text and Picture Option Grid will be available to clinicians and patients on paper pads and integrated into the EHR, leading to a website where the clinician can select the uterine fibroid Option Grid and choose any number of treatment options specific to the patient to generate the comparison table. The online version also offers the clinician a standard phrase to document the presentation of the Option Grid to patients. It can be copied and pasted into the EHR, and the tool can also be sent via the patient portal or by email to the patient. The content and layout of the online Option Grid will be maintained to match that of the text and Picture Option Grid versions for the duration of the project period. The different versions provide clinicians with options, so they can choose the format that best suits their workflow. Both versions will be available in English and Spanish (see “Translation procedure” section for details).

Option Grid PDAs, in any of the different formats, are designed as catalysts for discussion between patients and clinicians to compare options and come to agreement about the best treatment choice.

### Translation procedure

The text and Picture Option Grid used in this study will be translated into Spanish using an adapted version of the Translate, Review, Adjudicate, Pretest, Document (TRAPD) translation procedure [27, 28]. Our translation approach will include four main stages: (i) Two suitably qualified native speakers of the target language create independent translations of the original text; (ii) a bilingual reviewer (who is a native speaker of the target language) compares the original text, translation 1, and translation 2 and either selects the preferred translation or produces a third translation that builds on the previous two; (iii) the bilingual reviewer meets with the project team to review and reconcile translations by consensus; (iv) the resulting translation is tested via cognitive debrief interviews with a small sample of patients fluent in the target language. Cognitive interview participants will each be offered a $10 honorarium.

### Outcomes and data collection

#### Primary outcome measure

The primary outcome measure is the number of eligible patients who receive the uterine fibroid Option Grid (three formats available).

**Fig. 3:**
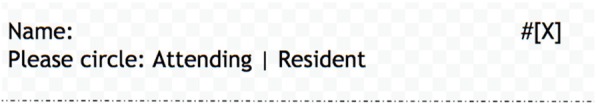
Paper-pad mockup to identify the number of eligible patients receiving the uterine fibroid Option Grid in the clinic

Eligible patients will be identified in advance by the project team at each site through their outpatient scheduling systems. This process will enable us to determine the total number of patients who are eligible to participate in the study (the denominator). To calculate the number of patients who receive an Option Grid (numerator), we will provide each site with a box filled with text and Picture Option Grid paper pads. Pads will be available in every consultation room within each sites’ obstetrics-gynecology clinic. To use a paper-based Option Grid, the clinician will tear it from the pad and a numbered stub will remain where they will write their name and indicate if they are an attending clinician or a resident (see Fig. 3 for a mockup of the paper pad stub). The research assistant at each site will refer to the numbered stubs to document the number of patients who received an Option Grid on a weekly basis. The research assistant will keep a log of the numerator and denominator in an Excel file and will report the data back to the Dartmouth project staff on a weekly basis via the Qualtrics database.

For sites with EHR access to the online uterine fibroid Option Grid, we will derive weblog data from EBSCO Health including the date and time the tool was generated; this data will be incorporated into measurement of the primary outcome.

#### Secondary outcome measures

In addition to recording each site’s chosen implementation strategy and if/how it changes during the implementation and sustainability phases, we will collect the following secondary outcome measures (as outlined in Table 2):

- *Measuring Organizational Readiness for patient Engagement (MORE)***.** MORE is designed to measure a healthcare organization’s willingness and ability to effectively implement patient engagement in healthcare [29]. The measure contains 25 items that are each scored on a four-point Likert-type scale (1 = avoid/not important; 4 = definitely involve/very important) [29]. Data from the measure will be analyzed to identify barriers and facilitators to implementation at each site and tailor the implementation strategy accordingly.
  Each site PI will be asked to nominate 20–30 MORE recipients within their department. Dartmouth study staff will then email the online survey link to nominees in the 2 months preceding the pre-implementation phase at each site.
- *Attitudes toward Decision Aids fOr PatienTs (ADOPT).* ADOPT is a measure of patient attitudes and perceptions toward PDAs. Clinicians are asked to select one or more words that best describe(s) their attitudes to the potential use of PDAs from a pool of 10 words. It can be completed by clinicians who have, or have not, used PDAs.
  ADOPT will be administered to all participating clinicians at three time points: (i) the start of the pre-implementation phase, (ii) the end of the active implementation phase, and (iii) the end of the sustainability phase. The surveys will be distributed via email to participating clinicians using a secure web link.
- *Extent of shared decision making.* Observer OPTION-5 is a validated, observational measure. It is a five-item scale, with each item rated from “0” to “4” where “0” represents the absence of a shared decision-making specific competency and “4” represents optimal performance [30].
  A research assistant employed at each site will audio-record five clinical encounters in both the pre- and active implementation phases for each participating clinician that consents to audio-recording. In each phase, we will establish a recruitment day(s) each week where a research assistant will approach as many eligible patients as possible to seek consent to audio-record their visit. This will be repeated each week until five encounter recordings per clinician per phase are obtained (10 per clinician in total). In the active implementation phase, we will begin the audio-recording recruitment process described above 2 months into the phase to allow clinicians time to get comfortable and confident with the intervention prior to recording. We will consult with sites to determine if we will audio-record encounters that take place between residents and patients. We anticipate a patient consent rate of 50 to 60% [31].
- *Fidelity of intervention use*. The fidelity assessment checklist enables us to determine if an Option Grid was used, how and when it was delivered to the patient, whether or not the clinician made a treatment recommendation or elicited patient preference, and the identification of the final treatment decision [32]. The checklist will help us understand if clinicians are using the tools as intended following initiation.
  The checklist will be completed independently by two Dartmouth-based project staff while listening to the audio-recorded clinic visits.
- *NoMAD Normalization Process Theory (NPT) survey.* A 23-item instrument used for capturing the perspective of professionals who will be involved in implementing and using Option Grid in practice [33]. The instrument contains four sections that ask questions on the various facets of implementing and using the intervention such as the effect that it has on workflow, the work and resources needed to drive implementation, and critical appraisal of the Option Grid.
  A convenience sample of 10 stakeholders at various levels of the service delivery team (i.e., clinicians, medical assistants, nurses, coordinators, managerial and reception staff) from each site will have the opportunity to complete the NoMAD survey at two time points: (i) at the end of the pre-implementation phase and (ii) at the end of the sustainability phase. Participants will be asked to provide their name each time they complete the survey. Stakeholders can complete the survey during a site visit, or they will have the opportunity to complete it online after the project team at Dartmouth sends a secure WebLink via email.
- *Utility of Option Grid PDAs and clinician approach to implementation*. Semi-structured interviews, guided by NPT, will be conducted with clinicians to assess the utility of the Option Grid tools and how they implemented the intervention in practice.
  A member of the project team at Dartmouth will conduct semi-structured interviews in-person or via phone with a convenience sample of clinicians at each of the five clinical sites to determine the utility of the uterine fibroid Option Grid PDA and the barriers and facilitators to its integration in the clinic workflow at two time points: (i) at the end of the active implementation phase and (ii) at the end of the sustainability phase.

**Table 2:** Implementation sustainability outcome measures for patient outcomes

	Implementation phases		
	Pre	Active	Sustainability
Organizational readiness (Measuring Organizational Readiness for patient Engagement survey)	✓		
Clinician attitudes to PDAs (ADOPT: Attitudes toward Decision Aids fOr PatienTs survey)	✓	✓	✓
Percentage of eligible patients receiving intervention (primary outcome measure)	✓	✓	✓
Quality of shared decision-making process using Observer OPTION-5	✓	✓	
Utility of tools and approach (clinician interviews)		✓	✓
Normalization Process Theory: NOMAD Toolkit (interviews)	✓		✓
Intervention fidelity assessment using observer checklist		✓	
Patient outcomes			
Uterine Fibroid Symptom and Health-Related Quality of Life Questionnaire (UFS-QOL) symptom severity subscale (8-items)	✓	✓	
**collabo**RATE (three items)	✓	✓	
The Comprehensive Score for financial Toxicity (COST)—one selected item	✓	✓	
Resource utilization (ambulatory and hospital visits)	✓	✓	
Intended and received treatment	✓	✓	
Chew health literacy measure (one item)	✓	✓	
Patient demographics (email address, health insurance status, age, race, gender, and spoken language)	✓	✓	

Throughout the pre- and active implementation phases, eligible patients at each site will be invited by clinic staff to take a survey on a tablet computer following their clinical encounter (see Additional file 2 to view the patient survey). Clinic staff may provide the patient with the tablet computer or refer them to a kiosk located in the clinic that will contain the tablet. At the start of the survey, patients will be given the opportunity to view the study information and provide consent to participate in the study. The survey will collect the following patient-reported outcomes, in addition to the patient’s email address which will be used to send patients a WebLink to the follow-up survey 3 months after their first survey is completed. The follow-up survey can be completed on a personal computer or mobile device. We anticipate that approximately 30% of eligible patients will complete the post-encounter survey. The following is a list of patient-reported outcomes that we will be assessing:

- ***collabo****RATE****.*** **collabo**RATE is a three-item, patient-reported experience measure of how patients perceive the effort that clinicians make to achieve three core aspects of shared decision making: informing patients, eliciting preferences, and integrating preferences into decisions made [27, 34]. Patients answer each question on a scale of 0 to 9. The measure is scored by using the percentage of patients who give the highest possible score of 9 for each item (top score) [27, 34].
- *Uterine Fibroid Symptom and Health-Related Quality of Life Questionnaire (UFS-QOL) symptom severity subscale.* We will ask patients to complete only the symptom severity subscale portion of the 37-item UFS-QOL questionnaire. The eight-item subscale asks patients how distressed their symptoms have made them feel over the past 3 months [35]. Each item is scored on a Likert-type scale from “1” (none of the time) to “5” (all of the time) [35].
- *The Comprehensive Score for financial Toxicity (COST).* COST is a patient-reported outcome measure to assess financial toxicity in patients with cancer [36, 37]. The measure contains 11 items. Study stakeholders, including patient partners, felt that the majority of the items were not applicable for this study and wanted to minimize respondents’ burden; therefore, we opted to only use one item. The question reads: “I worry about the financial problems I will have in the future as a result of my illness or treatment”, and the response scale is from “0” (not at all) to “4” (very much).
- *Chew Health Literacy Measure.* Three-item validated measure of health literacy. The items are the following: How confident are you filling out medical forms by yourself? How often do you have someone (like a family member, friend, hospital/clinic worker, or caregiver) help you read hospital materials? How often do you have problems learning about your medical condition because of difficulty understanding written information? Patients who circle “extremely” or “quite a bit” on the Likert-type scale are considered to have high health literacy [38].
- *Resource utilization (ambulatory and hospital).* The follow-up survey will query eligible patients to self-report the number of outpatients (or visit to primary care clinician), inpatient, or emergency visits during the 3-month follow-up period.
- *Treatment choice.* Patients will identify their intended treatment choice in the post-encounter survey. We will also ask patients in the 3-month follow-up survey whether they have seen another clinician about another treatment, whether an Option Grid tool was used in that encounter, and to identify the final treatment option they selected, or will select if they proceed with scheduling the treatment intervention.
- *Patient demographics*. We will collect the patient’s email address, health insurance status, age, race, gender, and spoken language via the survey they complete on the tablet computer post-encounter.

### Procedure

The implementation procedure was guided by CFIR’s inner setting and its related constructs (see Additional file 3 for details).

#### Clinician consent

Dartmouth project staff met with clinicians at each site in January and February 2019 to introduce the project. Co-investigators and stakeholder partners at each site will help identify clinicians who are eligible to participate in the study prior to the start of their pre-implementation phase and ahead of their second site visit. Prior to the second site visit, the principal investigators at each site will receive an information sheet describing the aims and methodology of the study via email. They will disseminate the information sheets to eligible clinicians. Eligible clinicians who wish to participate can provide their written consent in-person during the project staff’s second site visit, email their consent forms to the project staff at their earliest convenience, or return them to project staff at each site. Clinicians can opt-in on the consent form to have a sample of 10 of their clinical encounters audio-recorded over the course of project. We will determine before the site visits if residents are eligible to have a sample of their encounters audio-recorded. Clinicians who opt to participate will be sent a secure web link to a survey via email, so they can provide their age, gender, and years of experience post-fellowship (or year(s) of residency).

#### Randomization

The five participating sites were randomized by the project staff’s statistician, who was blinded to the identity of the sites, using R statistical software.

#### Pre-implementation phase

Prior to the start of the pre-implementation phase, stakeholders will complete the MORE survey. At the start of the pre-implementation phase, participating clinicians at each site will complete the ADOPT survey to indicate their attitude toward PDAs. We will audio-record five clinical encounters for each consenting participating clinician, and at the end of the study phase, we will conduct interviews with a convenience sample of clinicians and staff at each site to inform their completion of the NoMAD survey (see data collection section for details).

The Option Grid will not be used during the pre-implementation phase; however, patients will still complete the survey (see patient outcomes section for details) following their clinical encounters to provide baseline data for each site.

#### Initiation on how to practice shared decision making using the Option Grid tools

The study interventions will be carefully described to participating clinicians at each site during an initiation session that will take place within a 2-month phase between the pre- and active implementation periods. The principal investigators (GE and M-AD) will introduce the concept of shared decision making and Option Grid PDAs to stakeholders at each site using webinars, video-conferences, and face-to-face coaching based on the “three talk model of shared decision making” [39, 40]. The format of the initiation will be adapted for each site, based on MORE survey results. Each site will determine which Option Grid PDA formats to implement (text, picture, and online versions). During the initiation phase, the Dartmouth project team will also provide each site with its clinic-level **collabo**RATE top score percentage from the pre-implementation phase as insight into baseline patient-rated shared decision-making performance.

#### Active implementation phase

During each site’s active implementation phase, the uterine fibroid Option Grid PDA will be incorporated into the routine clinic workflow according to a tailored implementation strategy, as described in the “intervention” section above. Each site will keep a weekly record of the number of eligible patients who received the Option Grid (primary outcome).

As in the pre-implementation phase, patients will be asked to complete a survey (1) immediately following the clinical encounter and (2) 3 months after the clinical encounter.

Two months into the active implementation phase, research assistants at each site will begin audio-recording clinical encounters until each participating clinician (who consents to audio-recording) has had five of their encounters recorded. The audio-recordings will be sent to the project team at Dartmouth using a secure and HIPAA-compliant platform (Dartmouth Sharepoint). Two independent raters will assess (using the Observer Option-5 measure and fidelity checklist) the extent to which clinicians involve patients in the decision-making process using Option Grid tools. At the end of the active implementation phase, clinicians will complete the ADOPT survey, and we will conduct semi-structured interviews, guided by CFIR and NPT, with a convenience sample of participating clinicians to determine the tools’ utility, and the barriers and facilitators of their use in practice.

#### Sustainability phase

Prior to the sustainability phase, we will provide text and Picture Option Grid PDAs to each site. Project staff will limit communication with clinical sites throughout this phase to prevent unintended influence on continued PDA use. At the end of the sustainability phase, we will measure the number of eligible patients who receive the uterine fibroid Option Grid and clinician attitudes. We will also inquire about *if* and *how* study teams plan to use the text or Picture Option Grids after our study has ended, and we will assess the utility of the intervention from varied stakeholder perspectives at each site.

With the goal of offering guideline developers expertise on how to integrate shared decision-making processes into their documentation, we will contact organizations responsible for clinical practice guidelines regarding management of uterine fibroids. We will share the Option Grid and the evidence summaries created for the development of the existing tools, as well as indicating the new PCORI-generated evidence that we have considered in developing the PDA [41]. These steps will take place over the duration of the implementation study.

### Statistical analysis

#### Power calculation

The primary analysis has an expected sample size of 2600 patients across 30 to 40 clinicians (an estimated 87 patients per clinician [*n* = 30]) at each of the five sites. Because the intervention varies within clinician and our statistical models include clinician random and site-fixed effects, the relevant clustering variable is clinician-project week. With 40 project weeks and at least 30 clinicians, clustering is likely to have minimal impact on the results. Therefore, for a binary dependent variable (e.g., whether the uterine fibroid Option Grid is used or whether the patient gives a top **collabo**RATE score) with a baseline proportion of 0.5, a symmetric two-sided 95% confidence interval of width of at least 0.048 will have an 80% coverage probability of containing the true effect of the intervention, i.e., attaining a confidence margin of 0.048. In the context of shared decision making, such a margin is small and below typical levels of precision obtained from randomized studies.

#### Data analysis

Patient-level data will be clustered by site and clinician. Patients will be assigned to a single phase based on the date of their first encounter with a clinician at the site during the project period. Instances in which a patient has a repeated visit in the pre- and active implementation periods will be recorded to enable sensitivity analyses accounting for repeated observations.

Regression analyses will compare quantitative outcome measures (including survey scores and Option Grid use) between the pre- and active implementation phases while adjusting for patient and clinician characteristics. The extent to which clinicians involve patients in decision-making for each recorded encounter will be assessed by analyzing the Observer OPTION-5 and Option Grid fidelity checklist data from each phase of the study. We will conduct a framework analysis, guided by NPT, of the clinician interview transcripts to determine the barriers and facilitators of Option Grid implementation. Completion of the NoMAD Normalization Process Theory survey, based on interviews with site stakeholders, will enable us to make across-site assessments of implementation sustainability.

### Data management and storage

All patient and clinician survey data will be stored in the Dartmouth Qualtrics database, a HIPAA-compliant web-based data management system. We will assign a unique study identification number to patient and clinician participants. All audio-recordings of clinical encounters and semi-structured interviews will be loaded by site-based project staff into Dartmouth’s HIPAA-compliant SharePoint system. Each recording will be labeled with a unique identification number. For statistical analysis, data will be downloaded from Qualtrics and stored on an encrypted hard drive owned by Dartmouth College. Stata software will be used for statistical analysis.

## Discussion

Our study maps the various routes to implementing PDAs in clinic workflow by studying timing and mode of delivery, available formats (online, text, and picture), and tailored implementation strategies determined by each organization’s readiness for patient engagement. This study also explores ways to overcome the barriers and facilitators to implementation from various stakeholder perspectives, guided by CFIR and NPT. Further, we will assess the impact of the implementation strategy on clinical and other relevant outcomes for women across socioeconomic strata. Our study aims to improve healthcare delivery for women with symptomatic uterine fibroids by empowering them, through the use of a PDA, to be more active in the decision-making process.

We anticipate that healthcare organizations who wish to use PDAs will use our findings to inform their implementation strategy. This work will help stakeholders identify *how* and *when* to best deliver these tools to patients. This study can help identify barriers to implementation of PDAs, enabling providers to proactively address such barriers. Reflecting on their workflow will enable site staff to understand how to potentially change their system to better identify eligible patients and deliver the tool in a minimally disruptive fashion. Findings can encourage clinicians in gynecology departments to use PDAs with their patients given the positive effects of these tools on communication and outcomes.

The use of two validated frameworks to guide each site’s implementation strategy is a strength of our study [21–24]. In addition, we are assessing implementation from various stakeholder perspectives using both qualitative (interviews) and quantitative (survey) methods. We are providing each site with flexibility, so they can determine how best to implement Option Grid based on their clinic workflow patterns and organizational readiness for this type of approach. A potential limitation is that the outer setting domain of the CFIR framework is not addressed as extensively as the other domains (see Table 1). However, the integration of new evidence into existing clinical practice guidelines (component 4 of our strategy) addresses, to an extent, the external policy and incentives construct embedded in the CFIR outer setting domain. A further challenge will be determining the best method to ensure that all eligible patients receive the uterine fibroid Option Grid. However, we are collaborating with principal investigators, who are all practicing clinicians, at each site to overcome this challenge.

We will take an active approach to disseminating study findings by engaging key stakeholders—patients, caregivers, community members, health professionals, and policy makers—in identifying the message and the means of communication to appropriate audiences [42]. Potential dissemination strategies may include the use of social media, lay press, and academic.

## Additional files

Additional file 1: The SPIRIT checklist reporting guidelines. (DOCX 21 kb)

Additional file 2: The survey to be completed by eligible patients post-clinical encounter. (DOCX 22 kb)

Additional file 3: CFIR’s inner setting and its related constructs which guided our implementation procedure. (DOCX 14 kb)

## Data Availability

Data sharing is not applicable to this article as no datasets were generated or analyzed during the current study.

## Abbreviations

CBPR: Community-based participatory research
CFIR: Consolidated Framework for Implementation Research
COST: The Comprehensive Score for financial Toxicity
NPT: Normalization Process Theory
PDAs: Patient decision aids
UFS-QOL: Uterine Fibroid Symptom and Health-Related Quality of Life Questionnaire
