# Supplementary material for: Implementation of the uterine fibroids Option Grid patient decision aids across five organizational settings: a randomized stepped-wedge study protocol
Source: Implement Sci. 2019 Sep 2;14:88. doi: 10.1186/s13012-019-0933-z (PMC6721118; doi:10.1186/s13012-019-0933-z)
Supplement: Supplementary file 2 — The survey to be completed by eligible patients post-clinical encounter. (DOCX 22 kb) [file 13012_2019_933_MOESM2_ESM.docx]

Additional File 2 The survey to be completed by eligible patients post-clinical encounter.

[CONSENT TEXT]

C1. Having read the text above, would you like to continue with this survey?

1 Yes

2 No

TEXT1. [**SKIP IF T1; INCLUDE AT T0**]Thinking about the appointment you have just had…

CR1. [**SKIP IF T1; INCLUDE AT T0**]How much effort was made to help you understand your health issues?

| No effort was made |  |  |  |  |  |  |  |  | Every effort was made |
| --- | --- | --- | --- | --- | --- | --- | --- | --- | --- |
| 0 | 1 | 2 | 3 | 4 | 5 | 6 | 7 | 8 | 9 |

CR2. [**SKIP IF T1; INCLUDE AT T0**]How much effort was made to listen to the things that matter most to you about your health issues?

| No effort was made |  |  |  |  |  |  |  |  | Every effort was made |
| --- | --- | --- | --- | --- | --- | --- | --- | --- | --- |
| 0 | 1 | 2 | 3 | 4 | 5 | 6 | 7 | 8 | 9 |

CR3. [**SKIP IF T1; INCLUDE AT T0**]How much effort was made to include the things that matter most to you in choosing what to do next?

| No effort was made |  |  |  |  |  |  |  |  | Every effort was made |
| --- | --- | --- | --- | --- | --- | --- | --- | --- | --- |
| 0 | 1 | 2 | 3 | 4 | 5 | 6 | 7 | 8 | 9 |

TEXT3. Listed below are symptoms experienced by women who have uterine fibroids. Please consider each symptom as it relates to your uterine fibroids or menstrual cycle. Each question asks how much distress you have experienced from each symptom in the last 3 months.

There are no right or wrong answers. Please be sure to answer every question by ticking the most appropriate box for you. If a question does not apply to you, please mark “not at all” as a response.

|  | **During the last 3 months, how distressed were you by...** | **Not at all** | **A little bit** | **Somewhat** | **A great deal** | **A very great deal** |
| --- | --- | --- | --- | --- | --- | --- |
| UFS1. | Heavy bleeding during your menstrual period? | 1 | 2 | 3 | 4 | 5 |
| UFS2. | Passing blood clots during your menstrual period? | 1 | 2 | 3 | 4 | 5 |
| UFS3. | Variation in the length of your menstrual periods? | 1 | 2 | 3 | 4 | 5 |
| UFS4. | Variation in the number of days between each menstrual period? | 1 | 2 | 3 | 4 | 5 |
| UFS5. | Feeling tightness or pressure in your pelvic area (lower part of the belly)? | 1 | 2 | 3 | 4 | 5 |
| UFS6. | Frequent urination during the daytime? | 1 | 2 | 3 | 4 | 5 |
| UFS7. | Frequent night-time urination? | 1 | 2 | 3 | 4 | 5 |
| UFS8. | Feeling tired? | 1 | 2 | 3 | 4 | 5 |

CH1. [**SKIP IF T1; INCLUDE AT T0**] How confident are you filling out medical forms by yourself?

5 Extremely

4 Quite a bit

3 Somewhat

2 A little bit

1 Not at all

CH2. [**SKIP IF T1; INCLUDE AT T0**] How often do you have someone (like a family member, friend, hospital/clinic worker, or caregiver) help you read hospital materials?

5 All of the time

4 Most of the time

3 Some of the time

2 A little of the time

1 None of the time

CH3. [**SKIP IF T1; INCLUDE AT T0**] How often do you have problems learning about your medical condition because of difficulty understanding written information?

5 All of the time

4 Most of the time

3 Some of the time

2 A little of the time

1 None of the time

TEXT4. To what extent do you agree with the following statement?

|  |  | **Not at all** | **A little bit** | **Somewhat** | **Quite a bit** | **Very much** |
| --- | --- | --- | --- | --- | --- | --- |
| CST3. | I worry about the financial problems I will have in the future as a result of my illness or treatment. | 0 | 1 | 2 | 3 | 4 |

TX1. [**SKIP IF T1; INCLUDE AT T0**] At the moment, what option are you leaning toward to treat your uterine fibroids?

1 Watch and wait

2 Medicine with hormones

3 Medicine without hormones

4 Embolization (blocking blood flow to fibroids)

5 Endometrial ablation (destroy lining of uterus)

6 Myomectomy (surgery to remove fibroids)

7 Hysterectomy (surgery to remove uterus)

8 Other (please specify:___) [**TX1_7 SHORT OPEN-END**]

9 I am not sure

TX2. [**SKIP IF T0; INCLUDE AT T1**] Which treatment(s) have you received (or been scheduled to receive) for your uterine fibroids in the last three months? Please select all that apply. [**ACCEPT MULTIPLE RESPONSES**]

1 Watch and wait

2 Medicine with hormones

3 Medicine without hormones

4 Embolization (blocking blood flow to fibroids)

5 Endometrial ablation (destroy lining of uterus)

6 Myomectomy (surgery to remove fibroids)

7 Hysterectomy (surgery to remove uterus)

8 Other (please specify:___) [**TX1_7 SHORT OPEN-END**]

9 I am not sure

TX3. Do you remember seeing either of the following tools? Please select all that apply.

1 Picture Option Grid **[INCLUDE IMAGE][GO TO TX4 AT T0/TX]**

2 Option Grid table **[INCLUDE IMAGE] [GO TO TX4]**

3 I don’t remember seeing either tool **[GO TO TX7]**

TX4. **[SKIP IF T1; INCLUDE AT T0; ASK ONLY IF TX3=1 | TX3=2]**When did you receive the tool(s)? Please select all that apply. **[ALLOW MULTIPLE RESPONSES]**

**[INSERT IMAGES OF OG AND POG]**

1 Before my appointment

2 During my appointment

3 After my appointment

TX5. **[SKIP IF T1; INCLUDE AT T0; ASK ONLY IF TX3=1 | TX3=2]**How did you receive the tool(s)... (Please select all that apply.)

**[INSERT SIDE-BY-SIDE IMAGES OF OPTION GRID AND PICTURE OPTION GRID] [ALLOW MULTIPLE RESPONSES]**

|  |  |  | By mail | Through the online patient portal | Viewed on a clinic computer | Handed a paper copy in the clinic | Not sure |
| --- | --- | --- | --- | --- | --- | --- | --- |
| **[SHOW ONLY IF TX4=1]** | TX5a. | Before your appointment | 1 | 2 | 3 | 4 | 5 |
| **[SHOW ONLY IF TX4=2]** | TX5b. | During your appointment | **[DO NOT SHOW IN TX5b]** | 2 | 3 | 4 | 5 |
| **[SHOW ONLY IF TX4=3]** | TX5c. | After your appointment | 1 | 2 | 3 | 4 | 5 |

TX6. **[SKIP AT T1, INCLUDE ONLY AT T0; ASK ONLY IF TX3=1 | TX3=2]**Did you and your doctor use the tool(s) together to help you choose your treatment? **[INSERT SIDE-BY-SIDE IMAGES OF OPTION GRID AND PICTURE OPTION GRID]**

1 Yes

2 No

3 Not sure

TX7. **[ASK ONLY IF TX3=1 | TX3=2][INSERT IF T1: “**Thinking back to your appointment:**”]** Did the Option Grid tool(s) help you decide on a treatment for uterine fibroids?

**[INCLUDE SIDE-BY-SIDE IMAGES OF POG AND OG]**

1 Definitely helped

2 Probably helped

3 Did not make a difference

4 Probably did not help

5 Definitely did not help

TX8. [**SKIP IF T0; INCLUDE AT T1**] Since the first time you completed this survey three months ago, have you met with another doctor or clinician to discuss treatment options for your uterine fibroids? Do not include any scheduled surgeries or procedures.

1 Yes [**GO TO TX9**]

2 No [**SKIP TO D1**]

3 Not sure [**SKIP TO D1**]

TX9. **[ASK ONLY IF TX8=1]** Did you use an Option Grid tool (pictured below) during your later appointment(s)?

**[INSERT SIDE-BY-SIDE IMAGES OF OPTION GRID AND PICTURE OPTION GRID]**

1 Yes, the text-based Option Grid

2 Yes, the picture-based Option Grid

3 No, I did not use either tool

4 Not sure

D1. **[ASK ONLY AT T0; SKIP AT T1]**Which doctor did you see today? [**DROP-DOWN LIST**]

D2. What is your preferred email address? We will use this address to send your $20 Amazon gift card upon completion of this questionnaire [**INCLUDE AT T0**: “and to send you one more questionnaire like this one in three months”]. [**SHORT OPEN-END; VALIDATE EMAIL**]

D3. Do you have…

1 Commercial health insurance (through your employer, purchased on a government exchange, or purchased directly from an insurance company)

2 Medicare without supplemental insurance

3 Medicare with supplemental insurance

4 Medicaid

5 Other (please specify:___) [**D3_5 SHORT OPEN-END**]

6 Not sure

D4. What is your age?

1 18-30 years

2 31-40 years

3 41-50 years

4 51-60 years

5 61 years or older

6 Prefer not to say

D5. Are you… (Please select all that apply.) **[ACCEPT MULTIPLE RESPONSES]**

1 Female

2 Male

3 Non-binary

4 Transgender

5 Other

6 Prefer not to say

D6. What language do you speak at home?

1 English

2 Spanish

3 Other (please specify:___) [**D6_3 SHORT OPEN-END**]

D7. **[ASK ONLY AT T1; SKIP AT T0]**In the last three months, what treatments have you received or scheduled for your uterine fibroids? Please select all that apply. **[ALLOW MULTIPLE RESPONSES]**

**[LIST TREATMENT WORDING FROM FINAL OPTION GRID]**

TEXT5. In the last three months, how many times have you…

|  |  | None | 1 time | 2 times | 3 times | 4 or more times |
| --- | --- | --- | --- | --- | --- | --- |
| D8a. | Visited a doctor’s office for any reason related to your own health? | 1 | 2 | 3 | 4 | 5 |
| D8b. | Visited an emergency room or urgent care facility for any reason related to your own health? | 1 | 2 | 3 | 4 | 5 |
| D8c. | Stayed overnight in a hospital for any reason related to your own health? | 1 | 2 | 3 | 4 | 5 |
