# Supplementary material for: Implementation of the uterine fibroids Option Grid patient decision aids across five organizational settings: a randomized stepped-wedge study protocol
Source: Implement Sci. 2019 Sep 2;14:88. doi: 10.1186/s13012-019-0933-z (PMC6721118; doi:10.1186/s13012-019-0933-z)
Supplement: Supplementary file 3 — CFIR’s inner setting and its related constructs which guided our implementation procedure. (DOCX 14 kb) [file 13012_2019_933_MOESM3_ESM.docx]

Additional File 3 CFIR’s inner setting and its related constructs which guided our implementation procedure

| Construct | Short Description |
| --- | --- |
| Structural characteristics | The characteristics (age, maturity, size) of the organization |
| Networks and communication | The type and quality of the networks and related formal and informal communication occurring within the organization |
| Culture construct | Norms and values of the organization related to patient engagement in healthcare |
| Implementation climate | The organization’s capacity for change |
| Tension for change | The extent to which the organization does or does not tolerate the current situation |
| Compatibility | The fit between the intervention’s meaning and values and the individuals’ norms, values, as well as the intervention’s fit with existing workflows and systems |
| Relative priority | The perceived importance of the implementation within the organization |
| Organizational incentives and rewards | All relevant incentives (from extrinsic to salary raises and increased respect) |
| Goals and feedback | The extent to which goals are communicated to individuals within the organization, acted upon and fed back to them |
| Learning climate | The climate in which learning, change and successful implementation can occur |
| Readiness for implementation | The degree to which the organization is prepared, ready and committed to implement the intervention |
| Leadership engagement | The commitment, involvement and accountability of the organization’s leaders and managers |
| Available resources | The degree to which resources are available for implementation |
| Access to knowledge and information | Access to information about the intervention and to integrate it into routine practice |
